# Verification of chemistry reference ranges using a simple method in sub-Saharan Africa

**DOI:** 10.4102/ajlm.v5i1.404

**Published:** 2016-10-17

**Authors:** Irith De Baetselier, Douglas Taylor, Justin Mandala, Kavita Nanda, Christel Van Campenhout, Walter Agingu, Lorna Madurai, Eva-Maria Barsch, Jennifer Deese, Lut Van Damme, Tania Crucitti

**Affiliations:** 1 Institute of Tropical Medicine, Department of Clinical Sciences, STI Reference Laboratory, Nationalestraat, Antwerp, Belgium; 2 FHI 360, Durham, North Carolina, United States; 3 Scientific Institute of Public Health, Brussels, Belgium; 4 IMPACT Research and Development Organization, Kisumu City, Kenya; 5 Global Clinical and Viral Laboratory, Kwa-Zulu Natal, South Africa; 6 PathCare Bloemfontein, Quantum Building, Bloemfontein, South Africa; 7 Bill & Melinda Gates Foundation, Seattle, Washington, United States

## Abstract

**Background:**

Chemistry safety assessments are interpreted by using chemistry reference ranges (CRRs). Verification of CRRs is time consuming and often requires a statistical background.

**Objectives:**

We report on an easy and cost-saving method to verify CRRs.

**Methods:**

Using a former method introduced by Sigma Diagnostics, three study sites in sub-Saharan Africa, Bondo, Kenya, and Pretoria and Bloemfontein, South Africa, verified the CRRs for hepatic and renal biochemistry assays performed during a clinical trial of HIV antiretroviral pre-exposure prophylaxis. The aspartate aminotransferase/alanine aminotransferase, creatinine and phosphorus results from 10 clinically-healthy participants at the screening visit were used. In the event the CRRs did not pass the verification, new CRRs had to be calculated based on 40 clinically-healthy participants.

**Results:**

Within a few weeks, the study sites accomplished verification of the CRRs without additional costs. The aspartate aminotransferase reference ranges for the Bondo, Kenya site and the alanine aminotransferase reference ranges for the Pretoria, South Africa site required adjustment. The phosphorus CRR passed verification and the creatinine CRR required adjustment at every site. The newly-established CRR intervals were narrower than the CRRs used previously at these study sites due to decreases in the upper limits of the reference ranges. As a result, more toxicities were detected.

**Conclusion:**

To ensure the safety of clinical trial participants, verification of CRRs should be standard practice in clinical trials conducted in settings where the CRR has not been validated for the local population. This verification method is simple, inexpensive, and can be performed by any medical laboratory.

## Introduction

High prevalence rates for HIV, tuberculosis, malaria and several other infectious diseases are found in Africa.^1^ These high prevalence rates have led to an increasing number of clinical trials being conducted on the African continent. The majority of these trials aim to evaluate methods and interventions to reduce the burden of these diseases.^2,3^ To appropriately screen eligible participants and to detect and monitor possible toxicities related to the investigated products, accurate clinical laboratory reference ranges for the study population are required. These ranges were first introduced by Gräsbeck and Saris to describe fluctuations of blood parameter concentrations in well-characterised groups of individuals.^4,5^

Local clinical laboratories often rely on reference values established by the manufacturer or presented in a textbook, which are rarely specific for African populations.^6,7^ Previous studies have demonstrated that using laboratory reference ranges for haematology and biochemistry obtained from populations other than those under investigation have led to the possible exclusion of healthy participants and an over-reporting of adverse events.^6,8^ Thus, a good practice is to use reference ranges specific to the study population for appropriate and safe management of participants.^9^

In June 2009, we initiated a multi-centre phase III clinical trial to assess the safety of the antiretroviral combination tenofovir disproxil fumarate/emtricitabine (TDF/FTC) as pre-exposure prophylaxis for HIV among women in sub-Saharan Africa (FEM-PrEP trial).^10^ One of the primary objectives of the trial was to assess the safety of TDF/FTC in healthy women at high risk of acquiring HIV. The primary safety endpoints included confirmed grade 3 or grade 4 toxicity of aspartate aminotransferase/alanine aminotransferase (AST/ALT) and phosphorus and grade 2 or higher toxicity of creatinine during and up to four weeks after study product administration. Calculation of toxicity grades for the aforementioned parameters of interest required reference to the chemistry reference range (CRR).^10^ Abnormal renal and liver function values were exclusion criteria for study participation and were also protocol-required safety criteria for study drug interruption or permanent withdrawal. Accurate CRRs were therefore essential to guarantee the safety of the participants during the study and to provide adequate classification of adverse events.^11^

The analysers, the analytical methods and the study populations differed among the study sites in the FEM-PrEP trial, making the transferability of CRRs between sites inappropriate. In European and United States-based clinical trials, it is common practice to test all study samples in a central laboratory to limit test variation across study sites.^12^ However, in multi-centre trials in sub-Saharan African settings, the use of a central laboratory is not always recommended due to difficulties related to sample transport, instability of biochemical compounds and undesired delays in reporting of results.^13^

According to the Clinical and Laboratory Standards Institute (CLSI), each laboratory should determine its own laboratory reference limits, including the CRR.^14^ The CRR should be derived from a healthy population representative of the study population.^14^ However, determination of a CRR can be complex, time-consuming and expensive.^15^ In short, the CLSI guidelines recommend the establishment of CRRs with at least 120 reference individuals using a non-parametric ranking method or, as an alternative, a robust method with a minimum of 20 samples from qualified reference individuals when there are sample size constraints.

The majority of clinical laboratories collaborating in the FEM-PrEP trial had nationally- or regionally-established CRRs or used the manufacturer’s ranges prior to the study. Use of manufacturer-defined CRRs may not be appropriate for clinical trial target populations due to potentially important differences in socio-demographic characteristics, environmental context, malnutrition, dietary patterns, genetics, or infection with helminths or other parasites (such as malaria or schistosomiasis).^6,7,16,17,18,19^ Notably, recent reference studies conducted in Africa have identified differences within populations and sometimes within sub-groups,^20^ making the applicability of such reference ranges less likely. Ultimately, use of non-population-specific CRRs could compromise the scientific validity of clinical trial conclusions by under- or overestimating the severity of adverse events.

Our objective was to use a feasible and inexpensive method to verify previously-established CRRs and assess the impact of the final revised CRRs on the observed safety results in our phase III clinical trial.

## Methods

### Ethical considerations

The FHI 360 Protection of Human Subjects Committee (PHSC), institutional review boards at all study sites and applicable regulatory committees approved the study.

### Study design

Women were recruited at four different sites in Bondo, Kenya; Pretoria and Bloemfontein, South Africa; and Arusha, Tanzania from June 2009 to April 2011. Because of a decision on April 18, 2011 to close the study early due to futility, verification of the CRR was not finalised for the Arusha site and is therefore not addressed in this paper. Women had to be aged between 18 and 35 years, not pregnant, and in general good health to be included in the study. The CRRs were verified at each site using a subset of 10 screened participants who were negative for: HIV, hepatitis B virus, *Chlamydia trachomatis, Neisseria gonorrhoeae, Trichomonas vaginalis, Candida* spp. and bacterial vaginosis.

### Sample collection

All laboratory activities, including specimen transport, processing, testing, result reporting and storage, were conducted in accordance with Good Clinical Laboratory Practices. At each study site, serum was collected in a plastic uncoated serum separation tube at screening, at weeks 4, 12, 24, 36, 52 and 56, and when clinically indicated. Samples were immediately taken to the on-site laboratory and were processed within two hours of collection. Quantification of AST/ALT, phosphorus and serum creatinine was performed according to the procedures described by the manufacturer and documented in site-specific standard operating procedures. The study site in Bondo performed the chemistry testing on-site using VITROS DT II (Ortho-Clinical Diagnostics, Inc., Johnson & Johnson, Buckinghamshire, United Kingdom) until May 2010 and thereafter used the VITROS 250 instrument (Ortho-Clinical Diagnostics, Inc., Johnson & Johnson, Buckinghamshire, United Kingdom). Two private laboratories performed the chemistry analysis on the samples collected at the South African study sites. Both laboratories were ISO 15189 accredited, had excellent infrastructure and had collaborated previously in multi-centre clinical trials. The study site in Pretoria shipped serum samples daily for chemistry analysis in temperature-monitored cool boxes with ice packs to the Global Clinical and Viral Laboratory (GCVL; Durban, South Africa). Upon arrival, the samples were immediately analysed using the Synchron CX5 Beckman Chemistry Analyzer (Beckman Coulter, Inc., Fullerton, California, United States). The Bloemfontein site transported the serum samples every two hours to PathCare Laboratories, located five minutes’ drive from the study site. The samples were transported at ambient temperature and were analysed immediately upon arrival using the Synchron CX5 Beckman Chemistry Analyzer. All sample transportation was validated before implementation.

### Laboratory methods

After the study began, we verified the CRRs for AST, ALT, creatinine and phosphorus according to guidelines established previously by Sigma Diagnostics based on the biological variation of the analytes (no reference available). In brief, the following was performed at each study site. The ALT, AST, creatinine and phosphorus values of serum samples collected from 10 clinically-healthy participants obtained at screening were used to calculate a ‘patient mean’, after which the mean of the current reference range was determined.

Example: Manufacturer’s reference range for AST = 9 to 52 U/L:

$$\begin{array}{l} \text{Reference Range Mean}=\left(\frac{\text{Upper Limit}-\text{Lower Limit}}{2}\right)\\ \;\;\;+\text{Lower Limit}=\left(\frac{52-9}{2}\right)+9=30.5U/L \end{array}$$ [Eqn 1]

The patient mean was compared with the established reference range mean and the percent difference between the selected samples, and the established reference mean was calculated.

Example: AST Reference Range Mean = 30.5 U/L

Selected Patient Sample Mean = 23.4 U/L

$$\begin{array}{l} \%Deviation=\left(\left(\frac{\text{Selected Sample Mean}}{\text{Reference Range Mean}}\right)x100\right)-100\\ =\left(\left(\frac{23.4}{30.5}\right)x100\right)-100=23.3\% \end{array}$$ [Eqn 2]

The percent (%) deviation was compared with the tolerance limit listed in the Reference Range Deviation Tolerance Limits table (Table 1).

**TABLE 1:** Reference range verification tolerance limits according to Sigma Diagnostics.

Analyte	% Deviation limit	Analyte	% Deviation limit
Alanine aminotransferase	+/− 14%	Creatinine	+/− 10%
Albumin	+/− 8%	Glucose	+/− 8%
Alkaline Phosphatase	+/− 20%	Total Iron	+/− 14%
Amylase	+/− 20%	LDL / LDH	+/− 14%
Aspartate aminotransferase	+/− 14%	LD-1	+/− 20%
Total Bilirubin	+/− 14%	Magnesium	+/− 16%
Calcium	+/− 9%	Total Protein	+/− 8%
Chloride	+/− 4%	Triglycerides	+/− 16%
Total Cholesterol	NA	Blood Urea Nitrogen	+/− 6%
Cholesterol, HDL	+/− 20%	Uric Acid	+/− 12%
Creatine Kinase	+/− 20%	All Other Analytes	+/− 20%
Creatinine Kinase-MB	+/− 20%		

HDL, high-density lipoprotein; LD-1, lactate dehydrogenase isoenzyme 1; LDH, lactate dehydrogenase; LDL, low-density lipoprotein; NA, Not Applicable.

If the percent deviation was within the listed tolerance limits, the current CRR could be used and no further action was required. Cases in which the percent deviation exceeded the tolerance limit required collection of additional values to adjust the ranges. We determined *a priori* to collect an additional 30 values in case adjustment was required. The mean and standard deviation (SD) were calculated from the 40 representative values. A range of (mean – 3 SD) to (mean + 3 SD) was set from these data, and any values that fell outside these limits were eliminated. Afterwards, the mean and SD were calculated from the remaining values. The reference range was determined to be the mean ± 2 SD. In the event of changes in analyser or reagent/methodology, re-verification of the established CRR was done according to the above-mentioned procedures.

From all sites, 20 values per analyte were available permitting us to compare the applied verification method with the CLSI guidelines.^14^ According to CLSI, the CRR is accepted when at least 18 values fall within the original reported limits. If three to four results fall outside these limits, another 20 reference values should be obtained. If no more than two of these new values fall outside the CRR, the CRR is accepted, otherwise the CRR should be corrected using the CLSI guidelines.

### Statistical analysis

The freeware ‘Reference Value Advisor’ (RVA) was used to perform all calculations according to the CLSI.^15^ To assess the impact of the CRR verification and adjustment on the toxicity grading, we calculated chemistry grades using both the pre-existing and newly-established CRRs. The statistical analysis to assess the impact of CRR verification on the toxicity grading used the database of the FEM-PrEP trial.^10^ We included all women who were randomised, made at least one follow-up visit where chemistries were assessed, and did not return their entire product unused. We graded laboratory chemistry measurements at each site as grade 1, 2, 3 or 4 (for ALT, AST and creatinine) or grades 2, 3 or 4 (for phosphorus) according to the Division of AIDS (DAIDS) table.^10^ In addition to DAIDS guidance, the protocol specified that any creatinine value during follow-up which exceeded 1.5 times baseline be coded as grade 1, even if the absolute measurement was less than 1.1 times the upper limit of normal (ULN). The onset date of each abnormality was the date when the abnormality was first detected, regardless of when the highest grade level occurred. Likewise, individual abnormalities were considered ongoing until the values returned to normal, even if there was a partial decrease in grade. We included all measurements obtained prior to primary censoring dates (e.g. on or before a participant’s week 52 visit, her seroconversion visit, or an earlier discontinuation visit, whichever came first), irrespective of adherence to treatment regimen. We computed the total number of toxicity events that would have been missed using only the initial CRRs.

## Results

### Verification of CRR

Table 2 summarises the initial analytes’ reference ranges used by the study sites and the CRR after verification. Prior to the study, both South African laboratories were using laboratory-specific reference ranges or those established by a competent national authority, whereas the Kenyan study site was using manufacturer CRRs. Based on the verification results, we had to recalculate the CRR for AST at the Bondo site and that for ALT at the Pretoria site. The pre-existing phosphorus reference range did not require revision at any study site. The reference range for creatinine had to be adjusted at all three sites, mainly due to changes in methodology, reagent or standard. The need to adjust the CRR was checked using the Sigma method and CLSI guidelines. Results between both methods were in complete concordance except for ALT in Pretoria, which should not have been corrected according to CLSI.

Table 3 compares the Pretorian CRR obtained with the Sigma method and the non-parametric method as described by CLSI, based on the availability of 128 values from the Pretorian site.

**TABLE 2:** Initial and revised chemistry reference range values for each study site.

Site	Test	Analyser	CRR Initial†	CRR Final‡
Bondo	ALT	VITROS DT60/ VITROS 250	9–52 U/L	NA§
	AST		14–36 U/L	2–27 U/L
	Creatinine		62–106 µmol/L	45–99 µmol/L
	Phosphorus		0.81–1.45 mmol/L	NA§
Bloemfontein	ALT	Synchron CX5 Beckman	10–32 U/L	NA§
	AST		10–32 U/L	NA§
	Creatinine		60–100 µmol/L	38–72 µmol/L
	Phosphorus		0.80–1.40 mmol/L	NA§
Pretoria	ALT	Synchron CX5 Beckman	10–45 U/L	4–26 U/L
	AST		5–40 U/L	NA§
	Creatinine		60–110 µmol/L	36–84 µmol/L
	Phosphorus		0.80–1.55 mmol/L	NA§

ALT, alanine aminotransferase; AST, aspartate aminotransferase; CRR, Chemistry reference range; NA, not applicable.

† Initial refers to the CRRs that were first used by the laboratory;

‡ Final refers to the CRR after revision;

§ Tests that had no revised CRRs.

**TABLE 3:** Comparison of chemistry reference range from the Pretoria site obtained with the Sigma method and the Clinical and Laboratory Standards Institute method.

Site	Test	CRR Initial†	CRR Final‡ Sigma (*n* = 40)	CRR Final CLSI using RVA (*n* = 128)
Pretoria	ALT	10–45 U/L	4–26 U/L	6–34 U/L
	AST	5–40 U/L	NA§	13–29 U/L
	Creatinine	60–110 µmol/L	36–84 µmol/L	44–88 µmol/L
	Phosphorus	0.80–1.55 mmol/L	NA	0.84–1.53 mmol/L

ALT, alanine aminotransferase; AST, aspartate aminotransferase; CLSI, Clinical and Laboratory Standards Institute; CRR, Chemistry reference range; NA, not applicable; RVA, Reference Value Advisor freeware.^15^

† Initial refers to the CRRs that were first used by the laboratory;

‡ Final refers to the CRR after revision;

§ Tests that had no revised CRRs.

### Impact of revised CRR on number of laboratory toxicities

The grading of adverse events based on laboratory abnormalities was performed in accordance with the DAIDS grading table and therefore relied on the ULN. The final revised CRRs were applied from the time of establishment; previous adverse events were not re-graded during the study.

Table 4 provides the total number of toxicities found during the trial using the initial CRR versus the final reference ranges for AST, ALT and creatinine. In our settings, the CRRs became narrower after adjustment, with a corresponding lowering of the ULN. As a consequence, the overall number of laboratory toxicities that occurred during the trial was higher using the adjusted ranges as compared to the pre-existing ranges. For hepatic toxicity management, 25 grade 2 ALT, 13 grade 3 ALT, 19 grade 2 AST, and two grade 3 AST results would have been graded differently using the initial CRRs. According to the initial CRRs, there were no grade 2 creatinine toxicities; however, 14 grade 2 creatinine toxicities were identified using the newly-calculated ranges.

**TABLE 4:** Toxicity grades for AST/ALT and creatinine using initial versus final reference ranges.

Toxicity	Grades	Final CRR											
		Grade 0		Grade 1		Grade 2		Grade 3		Grade 4		Total	
		Tests	%	Tests	%	Tests	%	Tests	%	Tests	%	Tests	%
**ALT toxicity**													
Initial CRR	Grade 0	8170	95.1	206	2.4	0	0.0	0	0.0	0	0.0	8376	97.5
	Grade 1	0	0.0	144	1.7	**25**	**0.3**	0	0.0	0	0.0	169	2.0
	Grade 2	0	0.0	0	0.0	27	0.3	**13**	**0.3**	0	0.0	40	0.5
	Grade 3	0	0.0	0	0.0	0	0.0	3	0.0	0	0.0	3	0.0
	Grade 4	0	0.0	0	0.0	0	0.0	0	0.0	3	0.0	3	0.0
	Total	8170	95,1	350	4.1	52	0.6	16	0.2	3	0.0	8591	100.0
**AST toxicity**													
Initial CRR	Grade 0	8128	92.7	412	4.7	0	0.0	0	0.0	0	0.0	8540	97.4
	Grade 1	0	0.0	186	2.1	**19**	**0.2**	0	0.0	0	0.0	205	2.3%
	Grade 2	0	0.0	0	0.0	16	0.2	**2**	**0.0%**	0	0.0	18	0.2
	Grade 3	0	0.0	0	0.0	0	0.0	1	0.0	0	0.0	1	0.0
	Grade 4	0	0.0	0	0.0	0	0.0	0	0.0	2	0.0	2	0.0
	Total	8128	92.7	598	6.8	35	0.4	3	0.0	2	0.0	8766	100.0
**Creatinine toxicity**													
Initial CRR	Grade 0	8458	97.4	43	0.5	**1**	**0.0**	0	0.0	0	0.0	8502 %	(97.9
	Grade 1	0	0.0	167	1.9	**13**	**0.2**	0	0.0	0	0.0	180	2.1
	Grade 2	0	0.0	0	0.0	0	0.0	0	0.0	0	0.0	0	0.0
	Grade 3	0	0.0	0	0.0	0	0.0	1	0.0	0	0.0	1	0.0
	Grade 4	NA		NA		NA		NA		0	0.0	NA	
	Total	8458	97.4	210	2.4	14	0.2	1	0.0	0	0.0	8683	100.0

Abbreviations: ALT, alanine aminotransferase; AST, aspartate aminotransferase; CRR, Chemistry reference range; NA, not applicable.

†, Final refers to the CRR after revision; ‡, Initial refers to the CRRs that were first used by the laboratory; §, Tests that had no revised CRRs.

The laboratory abnormality frequency for ALT, AST, creatinine, and phosphorus between the two study groups (placebo vs. TDF/FTC) using the initial and final CRRs were compared and are presented in Table 5. Although the majority of the missed toxicities were grade 1, half of the participants with a grade 3 or grade 4 hepatic toxicity would have been misclassified, if management had been based on the initial CRRs. The impact of the revised creatinine reference ranges was less pronounced. However, all five cases of grade 2 creatinine toxicity would have been misclassified as a grade 1 using the initial CRRs.

**TABLE 5:** Laboratory abnormality frequency tables based on the initial versus final chemistry reference ranges.

Parameter	Abnormality	Placebo (*N* = 1033) Initial/final CRRs†	TDF/FTC (*N* = 1021) Initial/final CRRs†	Total initial/final CRRs†	
		Number of events	Number of events	Number of events	Total number and percentage missed using initial ranges
ALT	Grade 1	57/84	60/131	117/215	98 = 45.6%
	Grade 2	7/8	14/16	21/24	3 = 12.5%
	Grade 3	2/6	2/4	4/10	6 = 60.0%
	Grade 4	2/2	1/2	3/4	1 = 25.0%
	**Grade 3 or 4**	4/8	3/6	7/14	7 = 50.0%
AST	Grade 1	79/174	81/205	160/379	219 = 57.8%
	Grade 2	7/13	10/21	17/34	17 = 50.0%
	Grade 3	1/1	0/2	1/3	2 = 66.7%
	Grade 4	0/0	1/1	1/1	0 = 0.0%
	**Grade 3 or 4**	1/1	1/3	2/4	2 = 50.0%
Creatinine	Grade 1	54/67	81/85	135/152	17 = 11.2%
	Grade 2	0/2	0/3	0/5	5 = 100.0%
	Grade 3	0/0	1/1	1/1	0 = 0.0%
	Grade 4	0/0	0/0	0/0	0 = 0.0%
	**Grade 2 or higher**	0/2	1/4	1/6	5 = 83.3%
Phosphorus	Grade 2	215/215	203/203	418/418	0 = 0.0%
	Grade 3	43/43	50/50	93/93	0 = 0.0%
	Grade 4	0/0	0/0	0/0	0 = 0.0%
	**Grade 3 or 4**	43/43	50/50	93/93	0 = 0.0%

ALT, alanine aminotransferase; AST, aspartate aminotransferase; CRR, Chemistry reference range; TDF/FTC, tenofovir disproxil fumarate/emtricitabine.

† Initial refers to the CRRs that were first used by the laboratory. Final refers to the CRR after revision.

Table 4 presents the total number of toxicities and Table 5 tabulates the number of adverse events based on laboratory abnormalities. Table 5 only shows the highest grade of abnormality occurrence even if this occurred after seroconversion, whereas Table 4 only includes results obtained on or before the primary censoring date. When comparing Tables 4 and 5, an idiosyncrasy in ALT data is noted. One participant had a grade 1 ALT abnormality before her primary censoring date (i.e., seroconversion visit) but experienced grade 3 AST toxicity 24 weeks after seroconversion according to the old CRR. When the new CRRs were used, the latter toxicity was reclassified as grade 4.

There were no significant differences in the proportion of women experiencing toxicities in the TDF/FTC and placebo groups based on the initial CRR (results not shown). Using the revised CRR, however, a significantly higher percentage of women in the TDF/FTC group experienced ALT grade 1 or higher toxicities (*p* = 0.033),^10^ and there was also a trend toward a higher percentage of women in the TDF/FTC group experiencing grade 2 or higher AST toxicities (*p* = 0.069).

## Discussion

The CLSI guidelines recommend the establishment of CRRs with at least 120 reference individuals using a non-parametric ranking method or, as an alternative, a robust method with a minimum of 20 samples from qualified reference individuals when there are sample size constraints. It was not feasible in the FEM-PrEP trial to recruit reference individuals prior to the initiation of the trial, as the study sites were research centres that did not see routine patients. Therefore, we verified the existing CRRs using specimens collected at screening using the Sigma verification procedure. This method is simple, does not require statistical expertise, is less time-consuming, inexpensive and can easily be implemented by any laboratory. We also examined the impact of the new reference ranges on the toxicity grading. When looking at our data, a total of 9 ALT/AST grade 3 or higher, 5 serum creatinine grade 2 toxicities and many grade 1 toxicities would have been missed if the original CRRs were used.

Laboratories are essential for both the detection and prevention of diseases. In clinical trials, laboratories play a crucial role in endpoint measurement. In the FEM-PrEP trial, the laboratory safety endpoints were based on chemistry parameters to detect liver and kidney toxicities. There were no additional clinical or laboratory costs involved in the verification process since the chemistry tests were a required screening procedure. The major disadvantage of using specimens collected at screening was that study participants could be excluded or included erroneously through misclassification of toxicity grades during the time of CRR verification and adjustment. However, in FEM-PrEP there were no instances of discordant eligibility classification when applying the pre-existing versus final verified CRRs due to predefined inclusion criteria, which required that creatinine be < 1.5 mg/dL and hepatic function tests be < 2x ULN.

Immunohaematological reference ranges are now well defined in Asia and Africa, and different studies have reported the need for population-specific clinical chemistry reference ranges.^17^ Recently, reference value studies among women and/or men in different countries in sub-Saharan Africa have been conducted.^6,16,17,18,19^ Table 6 summarises CRRs established in African countries compared with our final revised CRR. Previous studies in Kenya confirmed our creatinine values,^13,16,17^ but overall our CRRs for AST/ALT were narrower than those previously reported. For the South African sites, our ranges were also more compressed than in previous studies but, to date, no comparable studies have been performed in South Africa. The narrower ranges are explained by the fact that we calculated the ranges in a specific age group and population, namely, clinically healthy women aged between 18 and 35 years who were at high risk of acquiring HIV infection.

**TABLE 6:** Comparison of chemistry reference ranges from the three study sites to those reported in previous publications.

Analyte	Unit		Bondo (Kenya)	Pretoria (South Africa)	Bloemfontein (South Africa)	Uganda^6^	Rwanda, Uganda, Kenya and Zambia^16^	Kenya^17^	Ghana^15^	Kenya^13^	Tanzania^7^
**ALT**	U/L	LLN	9	4	10	5.3	8	10.7	6	8.6	0
		ULN	52	26	32	39.9	61	61.3	51	47	44.9
**AST**	U/L	LLN	2	5	10	11.4	14	13.5	13	13.1	13.5
		ULN	27	40	32	28.8	60	48.5	48	38.1	35.2
**Creatinine**	µmol/l	LLN	45	36	38	44.2	47	52.4	47	51	40
		ULN	99	84	72	79.6	109	96.8	110	91	81
**Phosphorus**	mmol/L	LLN	0.81	0.80	0.80	0.81	ND	ND	0.8	ND	ND
		ULN	1.45	1.55	1.40	1.81	ND	ND	1.5	ND	ND
**Population**			♀ (18–35y)	♀ (18–35y)	♀ (18–35y)	♀ (18–56y)	♂ + ♀ (18–34y)	♀ (18–34y)	♀ (18–59y)	♀ (18–55y)	♀ (19–48y)
**Analyser**			VITROS 60/250	Synchron CX5 Beckman Chemistry Analyser	Synchron CX5 Beckman Chemistry Analyser	Roche Cobas Integra 400 plus Analyser	Vitalab Selectra E Clinical Chemistry Analyser	Roche Cobas Integra 400 plus Analyser	Vitalab Selectra E Clinical Chemistry Analyser	Roche Cobas Integra 400 plus Analyser	Roche Cobas Integra 400 plus Analyser
**Calculation**			Sigma	Sigma	Sigma	Wilcoxon	CLSI	Statistics	CLSI	CLSI	CLSI
**Year published**			2014	2014	2014	2008	2009	2011	2012	2008	2008

CLSI, Clinical and Laboratory Standards Institute; LLN, lower limit of normal; ND, Not Done; ULN, upper limit of normal.

### Limitations

Our study has several limitations. Ideally, verification of reference ranges should be conducted before trial initiation. It is also possible that the number of specimens (10) required for initial verification by this method were too few. For example, CLSI recommends a set of 20 reference specimens and replacement of outliers if necessary. We compared the applied method with the CLSI guidelines and obtained similar results, except for one parameter (ALT) in one study site (Pretoria) which should not have been corrected according to CLSI. As we had more than 120 reference values available (*n* = 128), we recalculated the ALT CRR using RVA^13^ with the CLSI method and noted good concordance with the Sigma method.

### Conclusion

We detected a large number of toxicities that would not have been identified using the pre-existing CRRs due to the decrease in the ULN for hepatic and renal parameters. Overall, we developed more population-appropriate CRRs that may have improved the clinical safety management of study participants. In conclusion, establishing local reference ranges is necessary to comply with the high-quality standards of Good Clinical and Laboratory Practices. Unfortunately, not all laboratories have the resources necessary to establish local reference ranges; therefore, verification of existing reference ranges offers a good alternative. Methods such as the former Sigma method or freeware including robust, transformation and non-parametrical methods can be applied on reference samples sets without additional costs and in the absence of sophisticated statistics by any laboratory performing chemical analysis.

## Trustworthiness

### Reliability

During several laboratory supervision visits, the correctness of the values that were used to verify the CRR was checked with the raw data. All laboratories worked according to good clinical laboratory practice guidelines and two laboratories were also ISO 15189 accredited.

### Validity

We report here on an easy method that uses 10 values to verify the Reference Ranges and that can be implemented in any laboratory without need for statistical expertise. We also show that determining the CRR before the start of a clinical trial is imperative to ensure that all toxicities found in the study are graded correctly. With this study, we also compared the Sigma method with the CLSI guidelines and obtained similar results. Our reported CRR are also within range with what has been previously found.
